# Herpes Simplex Virus Type 2 Is More Difficult to Neutralize by Antibodies Than Herpes Simplex Virus Type 1

**DOI:** 10.3390/vaccines8030478

**Published:** 2020-08-27

**Authors:** Christiane Silke Heilingloh, Christopher Lull, Elissa Kleiser, Mira Alt, Leonie Schipper, Oliver Witzke, Mirko Trilling, Anna-Maria Eis-Hübinger, Ulf Dittmer, Adalbert Krawczyk

**Affiliations:** 1 Department of Infectious Diseases, West German Centre of Infectious Diseases, University Hospital Essen, University of Duisburg-Essen, D-45147 Essen, Germany; Christiane.Heilingloh@uk-essen.de (C.S.H.); chri.lu@gmx.de (C.L.); Mira.Alt@uk-essen.de (M.A.); Leonie.Schipper@uk-essen.de (L.S.); Oliver.Witzke@uk-essen.de (O.W.); 2 Biopharmaceuticals QC Services, Virology & Contamination Detection, Boehringer Ingelheim, D-55216 Biberach, Germany; elissa.kleiser@boehringer-ingelheim.com; 3 Institute for Virology, University Hospital Essen, University of Duisburg-Essen, D-45147 Essen, Germany; mirko.trilling@uni-due.de (M.T.); ulf.dittmer@uni-due.de (U.D.); 4 Institute of Virology, Medical Faculty, University of Bonn, D-53113 Bonn, Germany; Anna-Maria.Eis-Huebinger@ukb.uni-bonn.de

**Keywords:** HSV-1, HSV-2, neutralizing antibodies

## Abstract

Infections with herpes simplex virus type 1 (HSV-1) and type 2 (HSV-2) are a global health burden. Besides painful oral or genital lesions in otherwise healthy subjects, both viruses can cause devastating morbidity and mortality in immune-compromised and immune-immature individuals. The latter are particularly susceptible to a disseminated, life-threatening disease. Neutralizing antibodies (NAb) constitute a correlate of protection from disease, and are promising candidates for the prophylactic or therapeutic treatment of severe HSV infections. However, a clinical vaccine trial suggested that HSV-2 might be more resistant to NAbs than HSV-1. In the present study, we investigated the antiviral efficacy of the well-characterized humanized monoclonal antibody (mAb) hu2c against HSV-2, in a NOD/SCID immunodeficiency mouse model. Despite the fact that hu2c recognizes a fully conserved epitope and binds HSV-1 and HSV-2 glycoprotein B with equal affinity, it was much less effective against HSV-2 in vitro and in NOD/SCID mice. Although intravenous antibody treatment prolonged the survival of HSV-2-infected mice, complete protection from death was not achieved. Our data demonstrate that HSV-2 is more resistant to NAbs than HSV-1, even if the same antibody and antigen are concerned, making the development of a vaccine or therapeutic antibodies more challenging.

## 1. Introduction

Herpes simplex viruses (HSV) type 1 and 2 are distributed worldwide, and belong to the most prevalent virus infections in humans. Global estimates indicate that more than 3.7 billion people under the age of 50 (>67% of the population) are currently infected with HSV-1 [1], and approximately 500 million people suffer from HSV-2 [2]. Although the vast majority of primary infections have a subclinical course, all infections with HSV-1 and HSV-2 inevitably result in lifelong latent persistence of the virus in sensory ganglia [3]. Recurrent HSV-1 or HSV-2 infections cause a broad range of symptoms, ranging from painful and irritating but self-limiting oral or genital lesions to severe disseminated and life-threatening diseases in immune-compromised patients, or newborns that became infected during or briefly after pregnancy or delivery [2,4,5,6]. Furthermore, infections of the eye may result in the irreversible impairment of the visual capacity or even cause blindness [7,8]. Antiviral drugs such as aciclovir (ACV) are available, but their application fails to eliminate the latent reservoir, and does not provide long-term protection from recurrent HSV diseases. Furthermore, long time prophylactic treatment of high-risk patients such as immunosuppressed individuals (e.g., AIDS patients and transplant recipients) with conventional antiviral drugs is limited by the emerging occurrence of drug resistance [9]. Even after 60 years of research and promising results from animal studies, there is no licensed vaccine available for the prevention or treatment of HSV-1 or HSV-2 infections [10]. The Chiron HSV vaccine trial using recombinant HSV-2 glycoproteins B and D (gB2/gD2) [11] and the GlaxoSmithKline (GSK) Herpevac trial, which used recombinant gD2 [12], were the largest clinical trials conducted so far. While the data suggest that vaccine-induced neutralizing antibodies are a correlate of anti-HSV immunity and a benchmark for an effective protective or therapeutic vaccine, both trials did not show efficacy against HSV-2 disease [13,14,15].

Potent and broadly neutralizing antibodies are good candidates for prophylaxis and the therapy of viral infections, including HSV-1 and HSV-2. Accordingly, the administration of polyclonal or monoclonal neutralizing antibodies prior or after infection with HSV-1 or HSV-2 conferred significant protection in various animal models [16]. Furthermore, maternal IgG was shown to be sufficient to prevent neonatal HSV-1 infections of the CNS, indicating that either vaccine-induced or passively administrated antibodies might be a promising treatment option to reduce morbidity and mortality [17]. These encouraging results demonstrate the impact of neutralizing antibodies for the prevention and treatment of HSV infections. However, while hyper-immunoglobulin preparations or monoclonal antibodies are approved for the prevention and treatment of other viral infections such as hepatitis B [18] or rabies [19], an approved immunotherapy for HSV-1 or HSV-2 is currently not available. Important hurdles for the development of such preparations are that clinical and animal studies indicate that (I) the antibody responses raised by vaccines differ between humans and small animal models such as mice or guinea pigs, and (II) that neutralizing antibodies appear to neutralize HSV-1 and HSV-2 with different efficacies [20].

Well-characterized monoclonal antibodies are a potent tool to investigate the protective effect of antibodies against HSV-1 and HSV-2. The humanized antibody mAb hu2c, which was developed in our lab, is among the most advanced HSV-targeting monoclonal antibodies [21]. The antibody is directed against a highly conserved epitope on the glycoprotein B (gB) of HSV-1 and HSV-2 [21]. The humanized antibody mAb hu2c shows equal binding affinity as the parental murine antibody mAb 2c [21], which binds both HSV-1 gB and HSV-2 gB, with an equal affinity in a nanomolar range [21,22]. The humanized antibody was further investigated in various animal models, and was highly protective against HSV-1 infections in the NOD/SCID mouse model for immune deficiency [21], and mouse models for ocular HSV-1 infections of the cornea [23] and retina [24,25]. Furthermore, the antibody was shown to neutralize drug resistant clinical HSV-1 and HSV-2 isolates in vitro. Due to these promising results, the antibody is currently under investigation in a clinical phase 2 trial in patients with chronic recurrent anogenital HSV infection (EudraCT Number: 2019-000880-26). However, initial cell culture experiments indicated that HSV-2 might be less susceptible to neutralizing antibodies than HSV-1 [20,22], raising the apparent question of whether the humanized antibody is also less effective against HSV-2 infection in vivo. To answer the question would provide important insights concerning the fundamental role of neutralizing antibodies in preventing HSV-1 and HSV-2 infections in vivo. In the present study, we evaluated the antiviral efficacy of this antibody and a polyclonal, HSV-1 and HSV-2 neutralizing IgG in vitro. Finally, we investigated the performance of mAb hu2c against HSV-2 in the NOD/SCID mouse model for immunodeficiency.

## 2. Materials and Methods

### 2.1. Ethics Statement

Animal experiments were performed in accordance with the German regulations of the Society for Laboratory Animal Science (GV-SOLAS) and the European Health Law of the Federation of Laboratory Animal Science Associations (FELASA). The protocol was approved by the North Rhine-Westphalia State Agency for Nature, Environment and Consumer Protection (LANUV) (permit number: G 995/08).

### 2.2. Animals

Female NOD/SCID mice, 6–8 weeks of age, were purchased from Charles River Laboratories (Charles River Laboratories, Sulzfeld, Germany), and maintained under specified pathogen free conditions.

### 2.3. Cells and Viruses

Vero cells (American Type Culture Collection, ATCC, CCL81, Rockville, MD, USA) were cultured in Dulbecco’s modified Eagle medium (DMEM, Life Technologies Gibco, Darmstadt, Germany), supplemented with 10% (v/v) fetal calf serum (FCS; Life Technologies Gibco), 100 U/mL penicillin and 0.1 mg/mL streptomycin at 37 °C and 5% CO_2_.

HSV-1 strain F, HSV-2 strain G, HSV-2 R6, HSV-1-ΔgE-GFP and HSV-2(333)-GFP reporter viruses were propagated in Vero cells and stored at −80 °C. HSV-1-ΔgE-GFP (further referred to as HSV-1 GFP) was kindly provided by H. Hengel (Institute of Virology, Freiburg, Germany), and originally published by Farnsworth et al. [26] HSV2(333)-GFP (further referred to as HSV-2 GFP) was generated using the same strategy as described for the HSV-2(333)-betaGal recombinant virus by Taylor et al. 2007 [27]. The virus was a gift from P. Spear (Northwestern University, Chicago, IL, USA) and kindly provided by B. Sodeik (Institute of Virology, Hannover, Germany).

Viral titers were determined by a standard endpoint dilution assay and calculated as 50% tissue culture infectious dose (TCID_50_)/mL, as previously described [28]. The clinical isolate HSV-2 R6 is resistant against ACV and was previously described, together with a panel of drug resistant clinical isolates obtained from patients undergoing bone marrow transplantation [21].

### 2.4. Antibodies

The humanized monoclonal antibody mAb hu2c and the murine counterpart mAb 2c were purified from serum-free SP2/0- or hybridoma-supernatants by protein A chromatography, as previously described [21,22]. Briefly, columns containing 1 mL of protein A agarose (Thermo Fisher Scientific, Waltham, MA, USA) were adjusted to pH = 7.4 (for mAb 2c purification) or pH = 8 (mAb hu2c purification) with 20 mL binding buffer (100 mM Na_2_HP0_4_, 100 mM NaCl, 10 mM EDTA; pH = 7.4 or pH = 8). Serum-free cell culture supernatants containing mAb 2c or mAb hu2c were passed through the columns, respectively. The columns were washed with 20 mL binding buffer, and bound antibodies were eluted with elution buffer (100 mM glycine, pH = 2.5). Acidic pH was immediately neutralized with a neutralization buffer (1 M Tris/HCl, pH = 7.5), and purified antibodies were dialyzed against phosphate-buffered saline (PBS). Finally, antibody concentration was measured using a NanoDrop 2000 spectrometer. Polyclonal antibody formulation Intratect was purchased from Biotest Pharma AG (Dreieich, Germany).

### 2.5. Virus Neutralization Assay

Neutralizing capacities of the antibodies were determined by endpoint dilution assay, as described previously [22,29]. Briefly, serial dilutions of a polyclonal human IgG (0 to 3333 nM) or mAb 2c (0–125 nM) were incubated with 100 TCID_50_ of HSV-1 F or HSV-2 G for 1 h at 37 °C, in DMEM supplemented with 2% FCS, 100 U/mL penicillin, and 0.1 mg/mL streptomycin. The antibody virus inoculum was applied to confluent Vero cell monolayers grown in 96-well microtiter plates, and the cytopathic effect (CPE) was scored after 48 h of incubation at 37 °C and 5% CO_2_. The antibody concentration required for reducing virus-induced CPE by 100% was determined as the complete neutralization titer.

### 2.6. IC_50_ Determination by Reporter Virus Based Neutralization Assay

Serial dilutions of a polyclonal human IgG (0 to 3333 nM) or mAb 2c (0–125 nM) were incubated with 100 TCID_50_ of HSV-1-ΔgE-GFP or HSV-2(333)-GFP for 1 h at 37 °C in DMEM, supplemented with 2% (v/v) FCS, 100 U/mL penicillin, and 0.1 mg/mL streptomycin. Afterwards, the antibody virus inoculum was applied to Vero cell monolayers grown in black microtiter plates with clear bottom, and a cytopathic effect (CPE) was scored after 48 h of incubation at 37 °C by fluorescence determination, using a Mithras LB 943 microplate reader (Berthold Technologies, Bad Wildbad, Germany). The antibody concentration required for reducing the virus-induced fluorescence signal by 50% was determined as 50% inhibitory concentration (IC_50_). The calculation of IC_50_ values was performed using GraphPad Prism (GraphPadPrism Software, La Jolla, CA, USA).

### 2.7. Animal Studies

The antiviral efficacy of the monoclonal antibody mAb hu2c was investigated in immunodeficient NOD/SCID mice, as described before [21]. Briefly, female NOD/SCID (NOD.CB17-Prkdcscid/J) mice (Charles River Laboratories) were pre-treated with medroxyprogesterone acetate (Depo-Provera, Pfizer, NY, USA) to facilitate HSV-2 infection [30]. Subsequently, mice were anesthetized by intraperitoneal injection of ketamine (100 mg/kg) and xylazine (20 mg/kg), and intravaginally infected with 1 × 10^6^ TCID_50_/20 µL ACV resistant clinical HSV-2 R6 isolate. The same viral load was used for a clinical HSV-1 isolate in a prior study [21]. Skin glue (Epiglu; Meyer-Haake Medical Innovations, Wehrheim, Germany) was applied to the vulva to prevent discharge of the virus. Mice were monitored daily concerning the occurrence of HSV-typical symptoms, such as loss of weight, mucosal lesions, and neurological disease. Mice displaying any of these symptoms were sacrificed immediately. The mice were initially divided into four groups (*n* = 10). The intravenous (i.v.) treatment with the humanized antibody mAb hu2c was performed at two distinct doses. One group was treated according to a protocol that we had established for the treatment of a HSV-1 infection in NOD/SCID mice [21]. These mice received 300 µg mAb hu2c intravenously (i.v.), at 24 h, 40 h, and 56 h after infection. Unfortunately, one of the mice deceased before the onset of treatment, and was excluded from the experiment (therefore: 300 µg mAb hu2c group *n* = 9). The second group (*n* = 10) received a four-fold higher amount of mAb hu2c during the initial phase of infection than the first group. The mice were treated six times every 8 h and received 600 µg mAb hu2c (i.v.) at 24 h, 32 h, 40 h, 48 h 56 h, and 64 h after infection. Two control groups were included (*n* = 10, respectively). These mice received either PBS (i.v., at 24 h, 40 h and 56 h post infection) or aciclovir at 50 mg/kg every 12 h intraperitoneally. The viral loads were determined from vaginal irrigations obtained on days 1, 2, 4, 6, and 8 after infection on Vero cells by endpoint dilution assay. Therefore, the vaginal mucosa was washed two times with 20 µL PBS. Serial dilutions (1:10) of the vaginal samples in DMEM supplemented with 2% (v/v) FCS, 100 U/mL penicillin, and 0.1 mg/mL streptomycin were incubated on Vero cells for 48 h. Plaque formation was assessed by light microscopy and the titers were calculated as 50% tissue culture infectious dose (TCID50)/mL, as previously described [28].

### 2.8. Statistical Analysis

**Figure 1:**
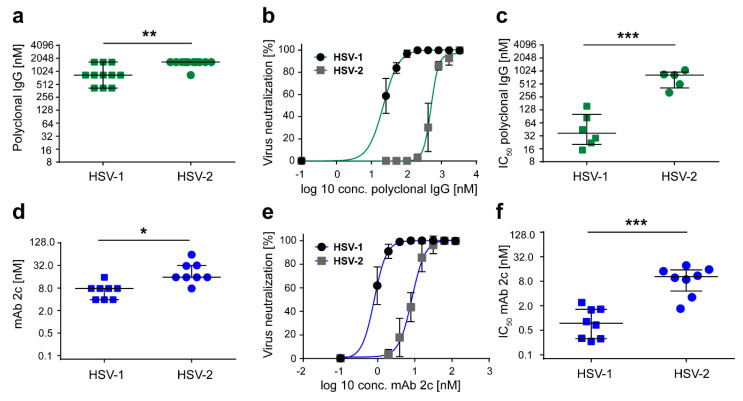
Herpes simplex virus (HSV)-2 withstands higher antibody concentrations before full in vitro neutralization is achieved. Neutralizing activity of (**a**) polyclonal IgG (0 to 3333 nM; *n* = 11) and (**d**) mAb 2c (0 to 125 nM; *n* = 8) against a defined viral load of 100 TCID_50_ HSV-1 F and HSV-2 G was determined. Data are displayed as median ± SD. (**b**,**c**,**e**,**f**) IC_50_ values of polyclonal IgG and mAb 2c against HSV-1 and HSV-2 infection were evaluated by endpoint dilution assays. Vero cells were infected with 100 TCID_50_ of HSV-1 GFP or HSV-2 GFP, and treated with serial dilutions of polyclonal IgG (0 to 33,330 nM; HSV-1 *n* = 6, HSV-2 *n* = 5) or mAb 2c (0 to 125 nM; *n* = 8). (**b**,**e**) Representative dose response curves are displayed as mean ± SD. IC_50_ values of (**c**) polyclonal IgG and (**f**) mAb 2c regarding HSV-1 GFP and HSV-2 GFP infection were calculated. Data sets were statistically evaluated using an unpaired *t*-test and significant changes are indicated by asterisks (* *p* < 0.05, ** *p* < 0.01, *** *p* < 0.001).

Statistical analysis was performed using GraphPad Prism 6 (San Diego, CA, USA). In vitro data shown in Figure 1 were statistically analyzed using an unpaired t-test. Viral titers of vaginal lavages were analyzed using Kruskal–Wallis test and Dunn’s multiple comparison post hoc test at day 8 post infection.

## 3. Results

### 3.1. Susceptibility of HSV-1 and HSV-2 to Neutralizing Antibodies In Vitro

We investigated the neutralization efficacy of polyclonal IgG toward HSV1 and HSV-2. For this purpose, serial dilutions (0 to 3333 nM) of polyclonal IgG (Intratect) were incubated with 100 TCID_50_ of HSV-1 or HSV-2 for 1 h, and added to confluent Vero cells. After 48 h of incubation, the cytopathic effect was scored by microcopy analysis. Notably, a significantly (*p* = 0.0428) higher concentration of polyclonal IgG was needed to achieve full neutralization of HSV-2 compared to HSV-1 (Figure 1a). While HSV-1 was completely neutralized at a concentration of 947.0 ± 149.6 nM polyclonal IgG, the full neutralization of HSV-2 was achieved at a concentration of 1591.0 ± 75.8 nM polyclonal IgG.

In a next step, the IC_50_ values of polyclonal IgG regarding HSV-1 and HSV-2 were determined (Figure 1b,c). For this purpose, an endpoint dilution assay was performed using GFP-expressing reporter viruses. In Figure 1b, representative dose response curves are displayed. The mean IC_50_ values of polyclonal IgG necessary for neutralizing HSV-2 (714.5 ± 133.0 nM) were one order of magnitude, and significantly higher (*p* = 0.002) than the corresponding IC_50_ values calculated for HSV-1 (58.1 ± 21.8 nM). These observations are in line with the results obtained from complete neutralization.

To exclude the possibility that the differences in neutralization efficacy of polyclonal IgG toward HSV-1 and HSV-2 are due to an unequal amount of neutralizing antibodies specific for these two viruses, or due to different binding affinities within the polyclonal IgG solution, we repeated the experiments using the well-characterized HSV-specific monoclonal antibody 2c (mAb 2c). MAb 2c is the murine counterpart of the humanized monoclonal antibody mAb hu2c. Both antibodies bind a highly conserved epitope on the HSV-1 and HSV-2 glycoprotein B, with an equal affinity in a nanomolar range [21]. The concentrations of mAb 2c required for complete neutralization of HSV-1 and HSV-2 as well as the IC_50_ values for HSV1 and HSV-2 were investigated using the endpoint dilution assays, as described above. The antibody was serially diluted from 0 to 128 nM. Complete neutralization was achieved at a concentration of 7.3 ± 1.4 nM and 24.5 ± 6.2 nM mAb 2c, for HSV-1 and HSV-2, respectively (Figure 1d). To fully neutralize HSV-2, a significantly higher amount of mAb 2c (*p* = 0.0007) was necessary compared to HSV-1. Furthermore, the determination of the IC_50_ values of mAb 2c regarding HSV-1 and HSV-2 also confirmed the results from the first experiment (Figure 1e,f). While HSV-1 showed 50% inhibition at a concentration of 1.0 ± 0.3 nM mAb 2c, a similar inhibition of HSV-2 required significantly (*p* = 0.0001) higher antibody concentrations of 10.4 ± 2.1 nM (Figure 1f). These data demonstrate that HSV-2 is less susceptible to neutralizing antibodies than HSV-1.

### 3.2. MAb hu2c Treatment Leads to a Prolonged Survival of HSV-2-Infected NOD/SCID Mice, but Fails to Prevent Lethality

**Figure 2:**
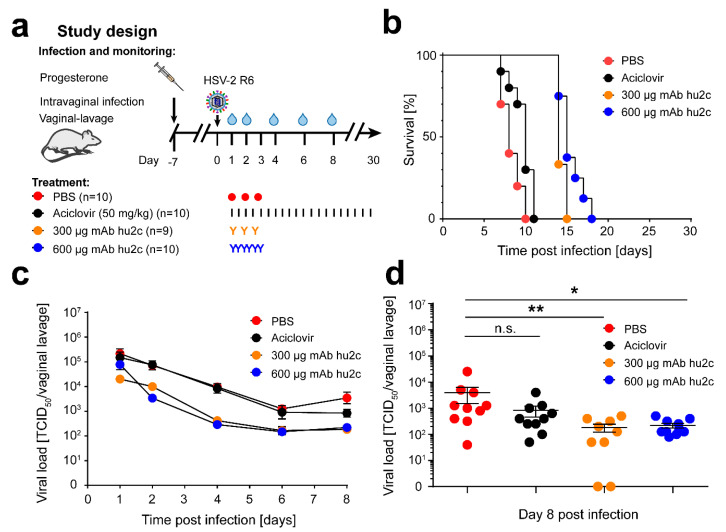
MAb hu2c treatment leads to a prolonged survival of HSV-2-infected NOD/SCID mice, but is not able to protect the mice from lethal HSV-2 infection. (**a**) Immunodeficient NOD/SCID mice (*n* = 10 per group, except 300 µg mAb hu2c group; *n* = 9) were intravaginally infected with 1 × 10^6^ TCID_50_/20 µL of the ACV-resistant clinical HSV-2 R6 isolate. Mice were treated with ACV (50 mg/kg; red circles) i.p. every 12 h, with mAb hu2c (300 µg, orange circles) i.v. at 24 h, 40 h, and 56 h after infection, or with mAb hu2c (600 µg, blue circles) i.v. at 24 h, 32 h, 40 h, 48 h, 56 h, and 64 h post infection. PBS administration (red circles) was used as mock treatment control and was applied i.p. every 12 h. (**b**) The figure shows survival curves of HSV-2-R6-infected NOD/SCID mice. (**c**) Virus titers of vaginal lavages were determined by endpoint dilution assay at indicated time points post infection. Data are displayed as mean ± SEM. (**d**) Virus titers of vaginal lavages were statistically evaluated using Kruskal–Wallis test and Dunn‘s multiple comparison post hoc test at day 8 post infection. Results are depicted as mean ± SEM, and significant changes are indicated by asterisks (* *p* < 0.05, ** *p* < 0.01) and non-significant changes as “n.s”.

To further investigate whether mAb hu2c can protect mice from a lethal HSV-2 infection, we examined the antiviral efficacy of this antibody in the NOD/SCID mouse model for immune deficiency. The study was performed using identical conditions as previously described for HSV-1, to allow for a fair and direct comparison of the neutralizing capacity of mAb hu2c against HSV-1 and HSV-2 in vivo [21]. In a previous study, we demonstrated that mAb hu2c treatment of NOD/SCID mice infected with a lethal dose of an ACV-resistant HSV-1 clinical isolate leads to viral clearance and protection from death [21]. NOD/SCID mice (*n* = 10 per group, except 300 µg mAb hu2c group; *n* = 9) were intravaginally infected with a viral load of 1 × 10^6^ TCID_50_ of an ACV-resistant clinical isolate HSV-2 R6, and intravenously treated with two distinct doses of mAb hu2c. The first group was treated i.v. with 300 µg mAb hu2c at 24 h, 40 h, and 56 h post infection, identical to the protocol published for HSV-1. The second group even received a fourfold higher dose of 600 µg mAb hu2c i.v. beginning at day 1 post infection, followed by injections every 8 h (24 h, 32 h, 40 h, 48 h, 56 h, and 64 h). Control mice received phosphate-buffered saline PBS as mock control, or received high dose treatment with ACV (50 mg/kg) every 12 h, starting at day 1 post infection. An overview of the animal study is depicted in Figure 2a. Figure 2b shows the survival rate of the HSV-2 R6-infected mice. All mice from the PBS- and aciclovir-treated groups died within the first 10 to 11 days, respectively. Treatment with mAb hu2c only prolonged the survival until day 15 (300 µg hu2c) and day 18 (600 µg hu2c). Surprisingly, and in sharp contrast to the previously published data regarding the HSV-1 infection [21], mAb hu2c treatment was incapable of protecting the HSV-2-infected mice from death, not even with the 4-fold higher antibody concentration. Furthermore, mAb hu2c treatment did not result in complete clearance of the virus from the vaginal mucosa. Figure 2c summarizes the data for the viral titers of vaginal lavages, at days 1, 2, 4, 6, and 8 post infection. The viral titers obtained for the mAb hu2c treated groups were significantly lower than the values obtained for the PBS and Aciclovir treated control groups. However, in contrast to the results obtained for HSV-1 infection, HSV-2 could not be eliminated. Taken together, our data demonstrate that HSV-2 is less susceptible to antibody immunotherapy than HSV-1, even when treated with a monoclonal antibody that binds its HSV-1- and HSV-2-derived antigen with equal affinity.

## 4. Discussion

In the present study, we evaluated the antiviral efficacy of the well-characterized monoclonal antibody mAb hu2c in vitro, and investigated the protective effect in the NOD/SCID mouse model for immune-deficiency. We demonstrated that polyclonal human immunoglobulin preparations and the monoclonal antibody neutralized HSV-1, with a significantly higher efficacy than HSV-2 in cell culture. Furthermore, the antibody showed protective effect in NOD/SCID mice that were infected with a lethal dose of an ACV resistant clinical HSV-2 isolate. However, the therapeutic effect of mAb hu2c was markedly lower in HSV-2-infected mice than in mice infected with an equal viral load of HSV-1 [21].

Neutralizing antibodies were shown to constitute a correlate of protection against HSV infections [31]. For instance, maternal antibodies that are transmitted by the mother to the offspring via the placenta are crucial for protection against a life-threatening HSV infection of the newborn, since mothers with a previous history of HSV rarely transmit the virus to their babies [32,33,34]. Thus, passive immunization with polyclonal hyperimmunoglobulin or monoclonal antibodies appears to be a treatment option for vulnerable individuals at high risk for a severe HSV infection. However, the establishment of a potent hyperimmunoglobulin or monoclonal antibody for clinical use against HSV-1 and HSV-2 infections appears challenging. The re-evaluation of former vaccine studies in animals and humans revealed that there are fundamental differences in the efficacy of antiviral antibodies toward HSV-1 and HSV-2 [12,15]. At this time, surprisingly, females who were immunized with a recombinant HSV-2 gD vaccine were found to be protected against genital HSV-1 infection, but not genital HSV-2 infection [12]. Accordingly, the serum antibodies of the vaccinated woman neutralized HSV-1 in cell culture more efficiently than HSV-2 [20]—despite the fact that the vaccine was intended to induce immunity against HSV-2. The reasons for the different efficacy of antibodies in neutralizing HSV-1 and HSV-2 are still unknown. In our hands, polyclonal human IgG also neutralized HSV-1 better than HSV-2. More interestingly, a well-characterized monoclonal antibody, mAb hu2c, which binds with an equal affinity to a highly conserved epitope on HSV-1 and HSV-2 glycoprotein B, neutralizes both viruses in a different manner. One explanation for the different neutralizing efficacy may be the role of the glycoproteins gC and gE in the immune evasion of HSV-1 and HSV-2. Both glycoproteins may affect the neutralizing effect of antibodies via targeting the IgG Fc-domain or shielding of other glycoproteins from neutralizing antibodies [35,36]. Furthermore, HSV-2 gC and gE were shown to protect the virus better from neutralizing antibodies than the HSV-1 gC and gE do. This might be one explanation why antibodies seem to be less effective against HSV-2. However, this effect is species dependent. Human IgGs are bound by gC and gE, while e.g., murine IgGs seemingly not [35]. In our hands, besides a polyclonal human IgG, a murine monoclonal antibody 2c neutralizes HSV-1 and HSV-2 with a different efficacy. Moreover, we demonstrated that also F(ab)_2_ fragments lacking the Fc-domain neutralize HSV-1 fourfold more efficiently than HSV-2 [22]. These findings strongly indicate that gC and gE dependent immune evasion of HSV is not the relevant reason why antibodies neutralize HSV-1 better than HSV-2.

To test if HSV-2 is also more difficult to neutralize in vivo, we analyzed the efficacy of the well-characterized humanized antibody mAb hu2c in mice. We conducted the study according to our previously described protocol with HSV-1 [21]. We used the same viral load of 1 × 10^6^ TCID_50_ for infection, and treated the mice with the humanized antibody accordingly three times with 300 µg mAb hu2c at 24 h, 40 h, and 56 h after infection. In a parallel group, mice were treated with a fourfold higher dose. The antibody (600 µg) was applied six times at every 12 h, starting at 24 h after infection. Mice infected with HSV-1 were almost completely protected from a lethal infection after receiving mAb hu2c three times at 300 µg, as previously described [21].

Surprisingly, all HSV-2-infected mice died, despite antibody treatment. With respect to a future clinical use of the antibody, the mice were infected with an aciclovir resistant isolate. As expected, ACV had no effect on the outcome of infection or the viral loads, as measured on day 8 after infection. Encouragingly, the lethal outcome of infection could significantly be delayed by systemic treatment with mAb hu2c in a dose-dependent manner. Furthermore, the viral loads measured from vaginal lavages were significantly lower compared to control treated mice. Although mAb hu2c could not mediate protection from a lethal outcome of infection, the antiviral effect of mAb hu2c against an ACV-resistant virus implies that mAb hu2c might be a promising candidate for immunotherapy of ACV resistant HSV2 infections. However, the dosage required to mediate the improvement of symptoms and protection from disease needs to be evaluated.

These results suggest that HSV-2 is less susceptible to neutralizing antibodies than HSV-1 in vivo. Our results are in line with findings from previous vaccine studies, where vaccine-induced antibodies were found to mediate protection from HSV-1, but not from HSV-2. The data provide evidence that HSV-2 is more difficult to neutralize than HSV-1, not only in vitro, but also in vivo. Previous studies demonstrated that HSV-2 is more virulent in mice or guinea pigs than HSV1 [37,38]. This might explain why it is challenging to develop a vaccine or an antibody-based immunotherapy that is effective against both HSV-1 and HSV-2. Further studies are needed to identify novel correlates of protection, crucial epitopes as potential targets for a vaccine, and novel antiviral antibodies that are capable of neutralizing both HSV-1 and HSV-2 in a similar manner.

## 5. Conclusions

In conclusion, we demonstrated that HSV-2 is more difficult to neutralize by antibodies such as the humanized antibody mAb hu2c than HSV-1 in vitro and in vivo. This finding needs to be considered for the future development of a vaccine or immunotherapy.

## 6. Patents

A patent application encompassing aspects of this work has been filed by the University of Duisburg-Essen and the University of Bonn, listing A.K. and A.M.E.H. as inventors.

## References

[1] Looker K.J., Magaret A.S., May M.T., Turner K.M.E., Vickerman P., Gottlieb S.L., Newman L.M. (2015). Global and Regional Estimates of Prevalent and Incident Herpes Simplex Virus Type 1 Infections in 2012. PLoS ONE.

[2] Looker K.J., Magaret A.S., Turner K.M.E., Vickerman P., Gottlieb S.L., Newman L.M. (2015). Global Estimates of Prevalent and Incident Herpes Simplex Virus Type 2 Infections in 2012. PLoS ONE.

[3] Nicoll M.P., Proença J.T., Efstathiou S. (2012). The molecular basis of herpes simplex virus latency. FEMS Microbiol. Rev..

[4] Bernstein D.I., Bellamy A.R., Hook E.W., Levin M.J., Wald A., Ewell M.G., Wolff P.A., Deal C.D., Heineman T.C., Dubin G. (2012). Epidemiology, Clinical Presentation, and Antibody Response to Primary Infection with Herpes Simplex Virus Type 1 and Type 2 in Young Women. Clin. Infect. Dis..

[5] Whitley R., Roizman B. (2001). Herpes simplex virus infections. Lancet.

[6] Kopp M., Aufderhorst U.W., Alt M., Dittmer U., Eis-Hübinger A.-M., Giebel B., Roggendorf M., Epple M., Krawczyk A. (2018). Induction of herpes simplex virus type 1 cell-to-cell spread inhibiting antibodies by a calcium phosphate nanoparticle-based vaccine. Nanomed. Nanotechnol. Boil. Med..

[7] Burrel S., Boutolleau D., Azar G., Doan S., Deback C., Cochereau I., Agut H., Gabison E.E. (2013). Phenotypic and genotypic characterization of acyclovir-resistant corneal HSV-1 isolates from immunocompetent patients with recurrent herpetic keratitis. J. Clin. Virol..

[8] Lau C.H., Missotten T., Salzmann J., Lightman S. (2007). Acute retinal necrosis features, management, and outcomes. Ophthalmology.

[9] Piret J., Boivin G. (2010). Resistance of Herpes Simplex Viruses to Nucleoside Analogues: Mechanisms, Prevalence, and Management. Antimicrob. Agents Chemother..

[10] Xu X., Zhang Y., Li Q. (2019). Characteristics of herpes simplex virus infection and pathogenesis suggest a strategy for vaccine development. Rev. Med. Virol..

[11] Corey L., Langenberg A.G.M., Ashley R., Sekulovich R.E., Izu A.E., Douglas J.J.M., Handsfield H.H., Warren T., Marr L., Tyring S. (1999). Recombinant glycoprotein vaccine for the prevention of genital HSV-2 infection: Two randomized controlled trials. JAMA.

[12] Belshe R.B., Leone P.A., Bernstein D.I., Wald A., Levin M.J., Stapleton J.T., Gorfinkel I., Morrow R.L.A., Ewell M.G., Stokes-Riner A. (2012). Efficacy Results of a Trial of a Herpes Simplex Vaccine. N. Engl. J. Med..

[13] Awasthi S., Friedman H.M. (2013). A Paradigm Shift: Vaccine-Induced Antibodies as an Immune Correlate of Protection Against Herpes Simplex Virus Type 1 Genital Herpes. J. Infect. Dis..

[14] Johnston C., Corey L. (2015). Current Concepts for Genital Herpes Simplex Virus Infection: Diagnostics and Pathogenesis of Genital Tract Shedding. Clin. Microbiol. Rev..

[15] Belshe R.B., Heineman T.C., Bernstein D.I., Bellamy A.R., Ewell M., Van Der Most R., Deal C.D. (2013). Correlate of Immune Protection Against HSV-1 Genital Disease in Vaccinated Women. J. Infect. Dis..

[16] Kimberlin D.M. (2010). Immunotherapy of HSV infections—Antibody delivery. Hum. Herpesviruses.

[17] Jiang Y., Patel C., Manivanh R., North B., Backes I.M., Posner D.A., Gilli F., Pachner A.R., Nguyen L., Leib D.A. (2017). Maternal Antiviral Immunoglobulin Accumulates in Neural Tissue of Neonates to Prevent HSV Neurological Disease. MBio.

[18] Adler R., Safadi R., Caraco Y., Rowe M., Etzioni A., Ashur Y., Shouval D. (1999). Comparison of immune reactivity and pharmacokinetics of two hepatitis B immune globulins in patients after liver transplantation. Hepatology.

[19] Aoki F.Y., Rubin M.E., Friesen A.D., Bowman J.M., Saunders J. (1989). Intravenous human rabies immunoglobulin for post-exposure prophylaxis: Serum rabies neutralizing antibody concentrations and side-effects. J. Biol. Stand..

[20] Awasthi S., Belshe R.B., Friedman H.M. (2014). Better Neutralization of Herpes Simplex Virus Type 1 (HSV-1) Than HSV-2 by Antibody from Recipients of GlaxoSmithKline HSV-2 Glycoprotein D2 Subunit Vaccine. J. Infect. Dis..

[21] Krawczyk A., Arndt M.A.E., Grosse-Hovest L., Weichert W., Giebel B., Dittmer U., Hengel H., Jäger D., Schneweis K.E., Eis-Hübinger A.M. (2013). Overcoming drug-resistant herpes simplex virus (HSV) infection by a humanized antibody. Proc. Natl. Acad. Sci. USA.

[22] Krawczyk A., Krauss J., Eis-Hübinger A.M., Däumer M.P., Schwarzenbacher R., Dittmer U., Schneweis K.E., Jäger D., Roggendorf M., Arndt M.A.E. (2010). Impact of Valency of a Glycoprotein B-Specific Monoclonal Antibody on Neutralization of Herpes Simplex Virus. J. Virol..

[23] Krawczyk A., Dirks M., Kasper M., Buch A., Dittmer U., Giebel B., Wildschütz L., Busch M., Goergens A., Schneweis K.E. (2015). Prevention of Herpes Simplex Virus Induced Stromal Keratitis by a Glycoprotein B-Specific Monoclonal Antibody. PLoS ONE.

[24] Bauer D., Alt M., Dirks M., Buch A., Heilingloh C.S., Dittmer U., Giebel B., Goergens A., Palapys V., Kasper M. (2017). A Therapeutic Antiviral Antibody Inhibits the Anterograde Directed Neuron-to-Cell Spread of Herpes Simplex Virus and Protects against Ocular Disease. Front. Microbiol..

[25] Bauer D., Keller J., Alt M., Schubert A., Aufderhorst U.W., Palapys V., Kasper M., Heilingloh C.S., Dittmer U., Laffer B. (2017). Antibody-based immunotherapy of aciclovir resistant ocular herpes simplex virus infections. Virology.

[26] Farnsworth A., Goldsmith K., Johnson D.C. (2003). Herpes Simplex Virus Glycoproteins gD and gE/gI Serve Essential but Redundant Functions during Acquisition of the Virion Envelope in the Cytoplasm. J. Virol..

[27] Taylor J.M., Lin E., Susmarski N., Yoon M., Zago A., Ware C.F., Pfeffer K., Miyoshi J., Takai Y., Spear P.G. (2007). Alternative Entry Receptors for Herpes Simplex Virus and Their Roles in Disease. Cell Host Microbe.

[28] Reed J.L., Muench H. (1938). A simple method of estimating fifty percent endpoints. Am. J. Epidemiol..

[29] Eis-Hübinger A.M., Schmidt D.S., Schneweis K.E. (1993). Anti-glycoprotein B monoclonal antibody protects T cell-depleted mice against herpes simplex virus infection by inhibition of virus replication at the inoculated mucous membranes. J. Gen. Virol..

[30] Parr M.B., Kepple L., McDermott M.R., Drew M.D., Bozzola J.J., Parr E.L. (1994). A mouse model for studies of mucosal immunity to vaginal infection by herpes simplex virus type 2. Lab. Investig..

[31] Hook L.M., Cairns T., Awasthi S., Brooks B.D., Ditto N.T., Eisenberg R.J., Cohen G.H., Friedman H.M. (2018). Vaccine-induced antibodies to herpes simplex virus glycoprotein D epitopes involved in virus entry and cell-to-cell spread correlate with protection against genital disease in guinea pigs. PLoS Pathog..

[32] Whitley R.J. (1994). Neonatal herpes simplex virus infections: Is there a role for immunoglobulin in disease prevention and therapy?. Pediatr. Infect. Dis. J..

[33] Prober C.G., Sollender W.M., Yasukawa L.L., Au D.S., Yeager A.S., Arvin A.M. (1987). Low Risk of Herpes Simplex Virus Infections in Neonates Exposed to the Virus at the Time of Vaginal Delivery to Mothers with Recurrent Genital Herpes Simplex Virus Infections. N. Engl. J. Med..

[34] Patel C., Backes I.M., Taylor S.A., Jiang Y., Marchant A., Pesola J.M., Coen D.M., Knipe D.M., Ackerman M.E., Leib D.A. (2019). Maternal immunization confers protection against neonatal herpes simplex mortality and behavioral morbidity. Sci. Transl. Med..

[35] Sprague E.R., Wang C., Baker D., Bjorkman P.J. (2006). Crystal Structure of the HSV-1 Fc Receptor Bound to Fc Reveals a Mechanism for Antibody Bipolar Bridging. PLoS Boil..

[36] Hook L.M., Huang J., Jiang M., Hodinka R., Friedman H.M. (2008). Blocking Antibody Access to Neutralizing Domains on Glycoproteins Involved in Entry as a Novel Mechanism of Immune Evasion by Herpes Simplex Virus Type 1 Glycoproteins C and E. J. Virol..

[37] Landry M.L., Myerson D., Bull C. (1992). Recurrent Genital Infection in the Guinea Pig: Differences between Herpes Simplex Types 1 and 2. Intervirology.

[38] Smith T.J., Ackland-Berglund C.E., Leib D.A. (2000). Herpes Simplex Virus Virion Host Shutoff (vhs) Activity Alters Periocular Disease in Mice. J. Virol..

